# Cell Proliferation on Polyethylene Terephthalate Treated in Plasma Created in SO_2_/O_2_ Mixtures

**DOI:** 10.3390/polym9030082

**Published:** 2017-02-25

**Authors:** Nina Recek, Matic Resnik, Rok Zaplotnik, Miran Mozetic, Helena Motaln, Tamara Lah-Turnsek, Alenka Vesel

**Affiliations:** 1 Jozef Stefan Institute, Jamova cesta 39, 1000 Ljubljana, Slovenia; rok.zaplotnik@ijs.si (R.Z.); miran.mozetic@ijs.si (M.M.); alenka.vesel@guest.arnes.si (A.V.); 2 Jozef Stefan International Postgraduate School, Jamova cesta 39, 1000 Ljubljana, Slovenia; matic.resnik@ijs.si; 3 National Institute of Biology, Vecna pot 111, 1000 Ljubljana, Slovenia; helena.motaln@nib.si (H.M.); tamara.lah@nib.si (T.L.-T.)

**Keywords:** polymer, polyethylene terephthalate (PET), SO_2_ plasma treatment, surface modification, wettability, X-ray photoelectron spectroscopy (XPS), atomic force microscopy (AFM), human umbilical endothelial cell (HUVEC) proliferation, MTT, toxicity, scanning electron microscopy (SEM)

## Abstract

Samples of polymer polyethylene terephthalate were exposed to a weakly ionized gaseous plasma to modify the polymer surface properties for better cell cultivation. The gases used for treatment were sulfur dioxide and oxygen of various partial pressures. Plasma was created by an electrodeless radio frequency discharge at a total pressure of 60 Pa. X-ray photoelectron spectroscopy showed weak functionalization of the samples’ surfaces with the sulfur, with a concentration around 2.5 at %, whereas the oxygen concentration remained at the level of untreated samples, except when the gas mixture with oxygen concentration above 90% was used. Atomic force microscopy revealed highly altered morphology of plasma-treated samples; however, at high oxygen partial pressures this morphology vanished. The samples were then incubated with human umbilical vein endothelial cells. Biological tests to determine endothelialization and possible toxicity of the plasma-treated polyethylene terephthalate samples were performed. Cell metabolic activity (MTT) and in vitro toxic effects of unknown compounds (TOX) were assayed to determine the biocompatibility of the treated substrates. The biocompatibility demonstrated a well-pronounced maximum versus gas composition which correlated well with development of the surface morphology.

## 1. Introduction

Polymer materials often require surface modification to achieve the best results in particular applications [1,2,3,4,5,6,7]. Different methods can be used for modification of the polymer surface properties, including chemical treatments, irradiation with photons, irradiation with ion or electron beams and treatment by gaseous plasmas created by electric discharges (corona, dielectric barrier discharge, glow discharge, etc.). Among all of these methods, plasma treatments remain the most prominent techniques for surface modification [8,9,10,11,12,13,14]. By appropriate selection of the type of discharge, treatment conditions, and working gases, it is possible to graft different surface functional groups, to change surface morphology, and even to create nanostructures on the treated surface. This way the surface wettability can be changed from superhydrophilic to superhydrophobic finishes [15].

In biomedical applications, and many others like surface cleaning, sterilization, improving surface wettability, and adhesive properties, the best results are most commonly achieved by the use of oxygen-containing plasma. Especially in biomedical applications, oxygen plasma was found to improve hemocompatibility of polymer grafts by making the polymer surface antithrombogenic because of reduced platelet adhesion [16]. Furthermore, oxygen plasma also improves cell adhesion and proliferation [17,18]. Therefore, oxygen plasma is a good alternative to the other methods used to make surface antithrombogenic, such as chemical grafting or various coatings like gelatin, heparin, fucoidan, etc. [19,20].

It has been demonstrated that sulfonic functional groups can also act in the antithrombogenic manner [21,22]. Commonly, such surface finish was accomplished by chemical synthesis of polymers containing sulfonic groups or by using special sulfated coatings like fucoidan [23,24,25]. In only a few attempts the SO_2_ plasma treatment was utilized as an alternative technique. Yet, biocompatibility tests of oxidized sulfur groups revealed some contradictory results. Some authors have observed increased platelet activation (i.e., increased surface thrombogenic effect) [26,27], whereas the others have reported on decreased platelet adhesion and activation (i.e., increased surface anti-thrombogenic effect) [21,22,28]. Despite the scarce literature, it appears that SO_2_ plasma-treated surfaces do not always display the most optimal antithrombogenic properties, as the best results were obtained for sulfonated surfaces prepared by chemical synthesis.

Indeed, very few papers were published regarding investigated cell adhesion on polymer surfaces treated with SO_2_ plasma. The first report was provided by Klee et al. [29], who studied adhesion of human umbilical vein endothelial cells (HUVEC), as well as fibronectin adsorption to medical grade polyvinyl chloride treated with SO_2_ plasma. They observed better fibrinogen adsorption on SO_2_ plasma-treated surface. Cell proliferation was in direct correlation with the fibronectin adsorption. [29]. Another reason for good cell proliferation was also a water contact angle of 67° presenting moderate hydrophilicity of the surface which is regarded as optimal for cell proliferation [30]. In another paper Wang et al. investigated the adhesion of dog vascular smooth muscle cells to SO_2_ plasma-treated polybutylene succinate surface and again found improved cell adhesion and growth on plasma-treated surface compared to the untreated one [31]. Likewise, Gugala was investigating the proliferation of rat osteoblast cells grown on polylactide surface treated with plasma containing SO_2_/H_2_ mixture [32]. In his study, SO_2_/H_2_ plasma treatment proved to negatively affect the cell growth. These rare results require further research of biocompatibility properties of surfaces with grafted oxidized sulfur groups.

More studies exis regarding the determination of chemical properties of SO_2_ plasma-treated surfaces. SO_2_ plasma was used for surface modification of different materials including polyethylene (PE) [26,27,33], polyethylene terephthalate (PET) [33], polymethyl methacrylate [34], polyvinylchloride (PVC) [29], polytetrafluoroethylene (PTFE) [35], polypropylene (PP) [33,36], polyesterpoly-uretane [36], heptylamine [37], octadiene [37], clay (laponite) [38], and highly-oriented pyrolytic graphite (HOPG) [36]. In these studies even the polytetrafluoroethylene (PTFE), which is quite difficult to activate by plasma, showed promising results [35]. Among the papers reporting results on SO_2_ plasma treatment, it is worth mentioning the works performed by Holländer et al. [33], Siow et al. [37], and Fatyeyeva et al. [38].

Fatyeyeva et al. [38] have studied the influence of discharge parameters (plasma power, gas flow rate, and treatment time) on kinetics of grafting sulfur moieties onto the treated clay surface. They found that increasing the gas flow leads to a significant decrease of sulfur content on the substrate surfaces; however, the sulfur content increased by increasing power and treatment time. Siow et al. [37] performed a detailed XPS characterization of plasma-treated heptylamine and 1,7-octadiene polymers and their post-treatment aging. They reported on groups with a higher oxidation state to be more influenced by aging in air. Holländer et al. [33] has compared treatment of polymers in pure SO_2_ plasma with those treated in a mixture of SO_2_ with oxygen and hydrogen. They found formation of highly oxidized sulfur groups in SO_2_ + O_2_ mixture, whereas in SO_2_ + H_2_ mixture sulfides prevailed. In pure SO_2_ plasma, both sulfides and highly-oxidized groups were observed. The effects of treatment conditions (discharge power and gas flow rate) were studied as well. At higher powers and flow rates more sulfur was detected on the surface. Furthermore, low plasma power lead to producing more sulfur in a low oxidation state, whereas a high flow of SO_2_ and a high power favored the formation of highly-oxidized species. 

Although there are discrepancies between different reports it can be summarized that SO_2_ plasma causes functionalization of polymers with sulfur groups, preferentially in the form of sulfites, however, the influence of such surface finish on proliferation of biological cells is still not understood well. In this study we systematically investigated the influence of SO_2_ plasma, as well as SO_2_/O_2_ mixtures on PET surface modification. PET is a material commonly used for artificial body implants like vascular grafts. To make PET surface biocompatible it should exhibit good endothelialization. The final goal was thus to create a biocompatible surface using the mixture of SO_2_/O_2_. To the best of our knowledge, no other group has done such work. SO_2_ plasma was chosen to mimic heparin, which is a commonly used coating on commercial vascular grafts for improving biocompatibility. Therefore, surface chemistry, morphology, and wettability of plasma-treated PET surfaces, as well as cell adhesion and surface toxicity were investigated.

## 2. Materials and Methods 

### 2.1. Plasma Treatment

**Figure 1:**
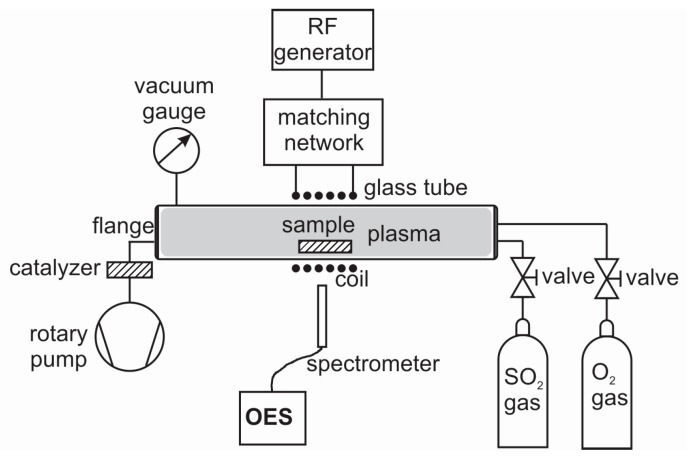
Schematic diagram of the experimental system.

Biaxially-oriented polyethylene terephthalate (PET) from Goodfellow Ltd. (Huntingdon, UK) was used as the substrate. The polymer foil with a thickness of 0.125 mm was cut to small square pieces with a size of 1 × 1 cm^2^. The samples were treated in a quartz-glass discharge tube presented schematically in Figure 1. The tube was 80 cm long and 4 cm in diameter. The discharge tube was pumped with a rotary pump operating at a nominal pumping speed of 80 m^3^ h^–1^. The base pressure was below 1 Pa. A mixture of SO_2_ + O_2_ was leaked to the experimental system on the other side as shown in Figure 1. The total pressure was set to 60 Pa where a maximum dissociation occurred, as measured by a catalytic probe. The ratio of SO_2_/O_2_ was varied. The purity of gases (which were supplied by Messer, Bad Soden, Germany) was 99.999 vol % and 99.98 vol % for O_2_ and SO_2_, respectively. A coil of six turns was mounted in the center of the Pyrex tube as shown in Figure 1. Plasma was created by a radio frequency (RF) generator coupled to the coil via a matching network. The generator operated at the standard frequency of 13.56 MHz and its nominal power was set to 150 W. Under such discharge conditions the plasma was sustained in the E-mode. Samples were treated in plasma for 30 s to allow for surface saturation. After the treatments they were characterized using atomic force microscopy (AFM), X-ray photoelectron spectroscopy (XPS), and water contact angle measurements (WCA).

### 2.2. Plasma Characterisation

Plasma was characterized using optical emission spectroscopy (OES) [39,40,41]. OES measurements were performed in a quartz tube with a 16-bit Avantes AvaSpec 3648 fiber optic spectrometer (Avantes Inc., Louisville, CO, USA). A nominal spectral resolution was 0.8 nm and spectra were recorded in the range from 200 to 1100 nm. The combined deuterium tungsten reference light source was used to determine the spectral response of the spectrometer. The measured OES spectra were calibrated with this spectral response. The integration time used to record OES spectra was 2 s.

### 2.3. Atomic Force Microscopy (AFM) Measurements

An AFM (Solver PRO, NT-MDT, Moscow, Russia) was used to characterize the topology of the samples. All measurements were performed in tapping mode using ATEC-NC-20 tips (Nano and More GmbH, Limerick, Ireland) with a resonance frequency of 210–490 kHz and force constant of 12–110 Nm^−1^. The surface roughness was calculated from AFM images taken over an area of 2 × 2 µm^2^ and 5 × 5 µm^2^ using the program Spip 5.1.3 (Image Metrology A/S, Hørsholm, Denmark). Surface roughness was expressed in terms of average roughness (Ra).

### 2.4. Characterisation by X-Ray Photoelectron Spectroscopy (XPS)

XPS characterization of polymer samples was performed to determine their chemical composition after plasma treatment using an XPS (a model TFA XPS Physical Electronics, Chanhassen, MN, US). The samples were excited with monochromatic Al Kα_1,2_ radiation at 1486.6 eV over an area with a diameter of 400 µm. Photoelectrons were detected with a hemispherical analyzer positioned at an angle of 45° with respect to the normal of the sample surface. XPS survey spectra were measured at a pass-energy of 187 eV using an energy step of 0.4 eV, whereas high-resolution spectra were measured at a pass-energy of 23.5 eV using an energy step of 0.1 eV. An additional electron gun was used for surface neutralization during XPS measurements. All spectra were referenced to the main C1s peak of the carbon atoms, which was assigned a value of 284.8 eV. The measured spectra were analyzed using MultiPak v8.1c software (Ulvac-Phi Inc., Kanagawa, Japan, 2006) from Physical Electronics, which was supplied with the spectrometer.

### 2.5. Contact Angle Measurements

The surface wettability was measured immediately after plasma treatment by determining the water contact angle (WCA) with a demineralized water droplet of volume 2 μL. Contact angles were measured by See System (Advex Instruments, Brno, Czech Republic). For each sample, five measurements were taken to minimize the statistical error. The contact angles were determined by the software supplied by the producer.

### 2.6. Cell Adhesion and Morphology Studies

Human umbilical endothelial cells (HUVEC; purchased from ATCC, Manassas, VA, USA) were cultured in a minimum essential medium (MEM; Sigma-Aldrich, Taufkirchen, Germany) supplemented with 10% fetal bovine serum (FBS; Sigma-Aldrich, Taufkirchen, Germany), 100 U penicillin, 1000 U streptomycin, 2 mM l-glutamine, and plated at density of 3000 cells/cm^2^. For the investigation of cell adhesion and morphology, the cells were seeded at a density of 2 × 10^4^ cells in 100 µL drop of medium on the upper side of the polymers (concentration: 2.55 × 10^4^ cells/cm^2^) and left for 1, 2, 3, 5, and 24 h to attach at 37 °C in a humidified atmosphere of 5% CO_2_. Cells were seeded onto modified polymer in duplicates for each time and plasma treatment condition.

Cell adhesion and morphology was assessed after 24 h of incubation (time allowed for cells to firmly attach on the surface) by scanning electron microscopy (SEM). Briefly, the polymer samples with the attached cells were fixed in 2% glutaraldehyde (Sigma-Aldrich, Taufkirchen, Germany) in phosphate-buffered solution for 5 min, followed by dehydration through an increasing gradient of ethanol and then vacuum dried by the critical point method. Finally, the samples were covered by a thin layer of gold and analyzed by SEM. For gold evaporation a PECS instrument (Model 682) from Gatan GmbH (München, Germany) was used. SEM analyses were performed using a JEOL JSM-840 Scanning Electron Microscope (JEOL, Tokyo, Japan).

#### 2.6.1. MTT (3-(4,5-dimethylthiazol-2-yl)-2,5-diphenyltetrazolium bromide) Assay

HUVECs were seeded and cultured in the same manner as for the cell adhesion and morphology investigation by SEM. The MTT-related colorimetric assay (EZ4U; Biomedica, Wien, Austria) was used to determine cell growth and viability, according to the manufacturer's instructions. The method is based on the fact that living cells are capable of reducing less-colored tetrazolium salts into intensely-colored formazan derivatives. This reduction process requires functional mitochondria, which are inactivated within a few minutes after cell death. Briefly, after 1, 2, 3, 5, and 24 h of HUVEC cell incubation on the differently modified polymer surfaces the medium was removed and the polymer samples were rinsed with phosphate buffer saline to remove for all non-attached cells. Then 200 μL of fresh Hanks’ Balanced Salt Solution (HBSS) (Sigma-Aldrich, Taufkirchen, Germany) mixed with the tetrazolium agent were added to each well with the polymer sample of the 24-well plate. After 1, 2, 3, 5, and 24 h of incubation, supernatants were transferred into 96-well plates and the absorbance was measured at OD 570/690 nm with SynergyTM HT Microplate Reader (Bio-TeK Instruments, Inc., Winooski, VT, USA). 

#### 2.6.2. In Vitro Toxicology Assay (TOX) 

The sulforhodamine B assay measures total biomass staining cellular proteins with sulforhodamine B. Cells were seeded on the samples at density of 3000 cells/cm^2^, tests were performed in duplicate, and each test included a blank containing complete medium without cells.

Fifty percent of (*w*/*v*) trichloroacetic acid (TCA, Sigma-Aldrich, Taufkirchen, Germany) solution and wash solution were prepared according to manufacturing instructions. Samples were removed from incubator into a laminar flow hood and cells were fixed by gently layering cold 50% (*w*/*v*) TCA solution on top of the growth medium. 96-well plates were incubated for 1 h at 4 °C and then rinsed with water several times to remove TCA solution, serum proteins, etc. Additionally, 0.4% sulforhodamine B solution was added in an amount sufficient to cover the culture surface area (~50% of the culture medium volume). Cells were then allowed to stain for 20–30 min. At the end of the staining period, the stain was removed and the cells were rinsed quickly with wash solution (1% acetic acid), until unincorporated dye was removed. Wash times were kept to a minimum to reduce desorption of protein-bound dye. After being rinsed, the cultures were air dried until no moisture was visible. The incorporated dye was then solubilized in a volume of sulforhodamine B assay solubilization solution (10 mM base solution, Sigma-Aldrich, Taufkirchen, Germany) equal to the original volume of culture medium and liberated from the cells. Cultures were allowed to stand for 5 min at room temperature, while pipetting up and down to enhance mixing of the dye. Absorbance was measured spectrophotometrically at a wavelength of 565 nm. Blank background optical density was measured in wells incubated with growth medium without cells. The background absorbance of multiwell plates was measured at 690 nm and subtracted from the measurement at 565 nm. An increase or decrease in the number of cells (total biomass) resulted in a concomitant change in the amount of dye incorporated by the cells in the culture. This indicated the degree of cytotoxicity caused by the test material. 

#### 2.6.3. Statistical Analysis

All the above experiments were performed in duplicate and independently repeated at least three times, unless otherwise stated. The results obtained are shown as the mean ± SE (standard error of the mean) for duplicates of cultures. Student’s *t*-test was used to test the effect different plasma modifications of PET have on the adhesion and metabolic activity of HUVECs and a value of *p* < 0.05 was considered significant.

## 3. Results and Discussion

### 3.1. Plasma Characterisation

**Figure 2:**
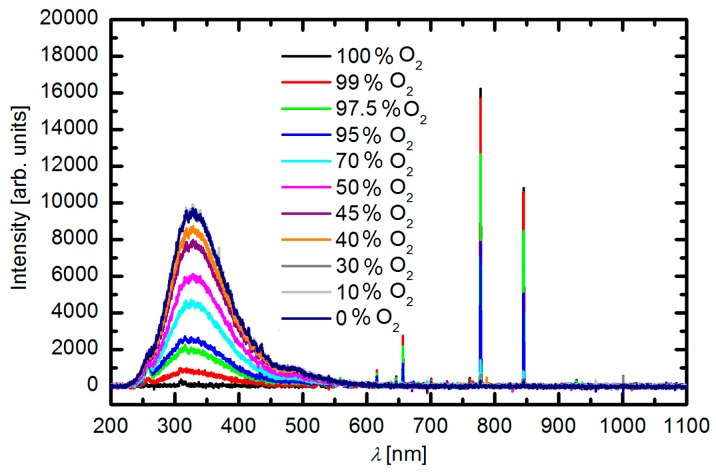
Spectra of plasma sustained in various gas mixtures. Parameter is the oxygen concentration in the SO_2_ + O_2_ mixture.

Gaseous plasma was characterized by optical emission spectroscopy and the resultant spectra for selected gas mixtures are presented in Figure 2. A large continuum in the ultraviolet (UV) range is attributed to the radiative relaxation of the SO_2_ molecule [42,43]. The large continuum is explained by the fact that the final state is the ground one, thus even electrons of moderate energy are capable of exciting SO_2_ molecules to the radiative states. The molecules are excited by electron impact and radiate in the broad range from UV A to UV C. Other spectral features are attributed to emissions from excited oxygen atoms. As usual, the most intensive is the line at 777 nm followed by the line at 845 nm. The radiation from the O-atoms is much weaker than the continuum because of the high threshold for excitation of the oxygen radiative states (approximately 11 eV).

**Figure 3:**
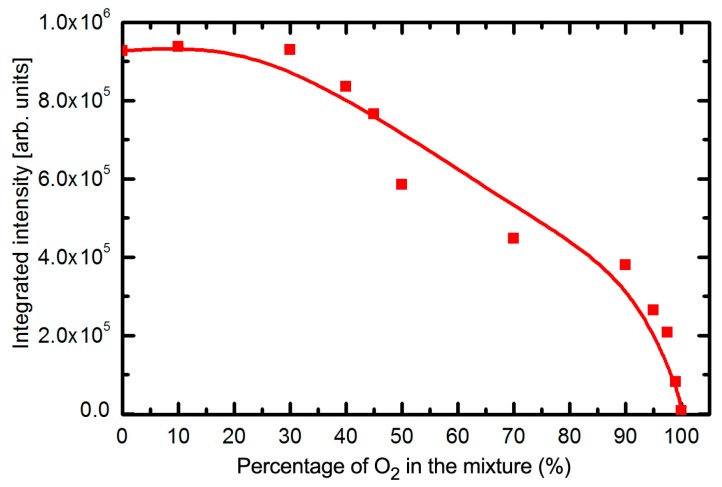
Integral radiation in the UV range versus oxygen concentration in SO_2_ + O_2_ mixture. Zero percent of O_2_ in the mixture refers to pure SO_2_ plasma, whereas 100% of O_2_ refers to pure oxygen plasma.

The intensity of radiation arising from SO_2_ molecules and O atoms depends on the concentration of gases in the gas mixture. Figure 2 reveals a gradual decrease of the continuum and increase of the atomic lines as the concentration of oxygen in the gas mixture increases. The integral radiation in the UV range as a function of the oxygen concentration in the SO_2_ + O_2_ mixture is plotted in Figure 3. The dots in this figure are measured points and the curve is the best fit. The integral intensity remains fairly unchanged up to the oxygen concentration of approximately 30 vol % and then decreases with the increasing concentration. Such behavior is explained by decreasing electrons’ density or their temperature because of the addition of substantial oxygen amounts. Namely, oxygen molecules represent additional channels for loss of electron energy and/or density because of dissociation and attachment onto oxygen atoms.

### 3.2. Surface Characterisation of Plasma-Treated Samples

**Figure 4:**
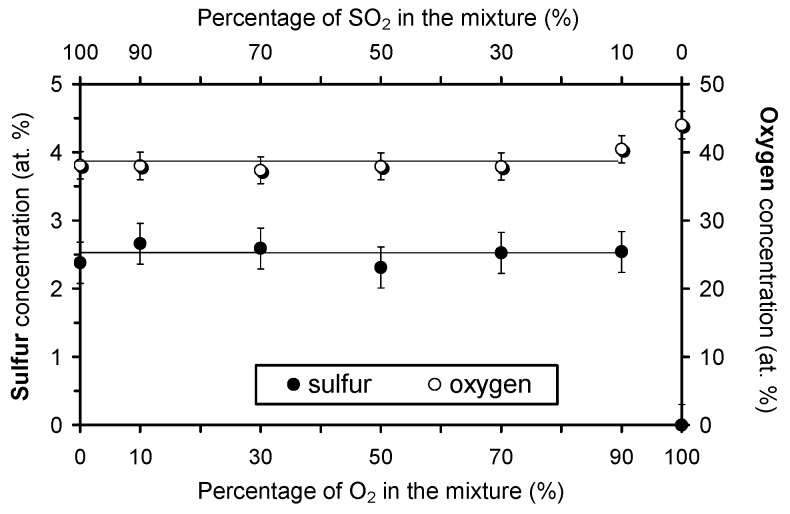
Concentration of sulfur and oxygen on the PET polymer treated in various SO_2_ + O_2_ mixtures. Zero percent of O_2_ in the mixture refers to pure SO_2_ plasma, whereas 100% of O_2_ refers to pure oxygen plasma.

Plasma-treated samples were characterized by XPS just after the treatment. The concentration of oxygen and sulfur on the polymer surface as extracted from the XPS survey spectra is presented in Figure 4. The concentration of sulfur is approximately 2.5 at % irrespective from plasma gas mixture. Obviously, the surface of the samples is saturated with sulfur groups upon a half-minute of plasma treatment what is sound with the results reported by other authors [33]. The reason for the saturation is the very high dissociation rate in our plasma and rather long treatment time. Additionally, the oxygen concentration on the polymer surface is not affected by adding oxygen to the gas mixture, because it remained close to the value for the sample treated in pure SO_2_ plasma (38 at %), except for the sample treated in a mixture with the highest oxygen content (10% SO_2_ + 90% O_2_) where it is somehow increased. The oxygen concentration obtained upon treatment in pure oxygen plasma is added to Figure 4 for comparison. For untreated PET sample, the oxygen concentration was 25 at % (not shown in the figure). 

**Figure 5:**
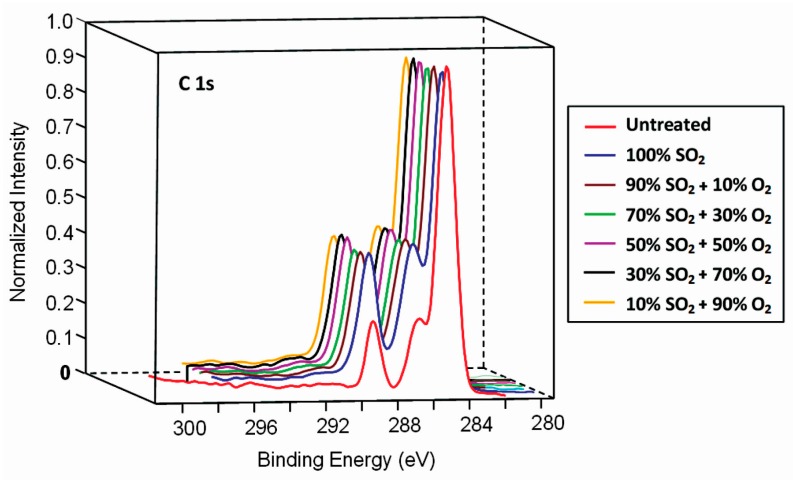
High-resolution C1s peak for samples treated at various SO_2_ + O_2_ mixtures.

**Figure 6:**
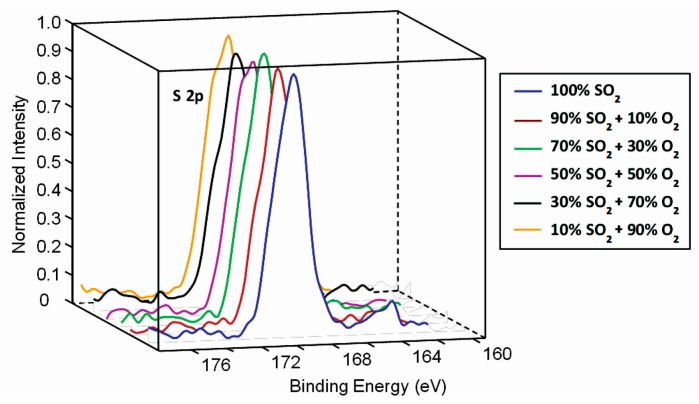
High-resolution S2p peak for samples treated at various SO_2_ + O_2_ mixtures.

The high-resolution XPS spectra of C1s and S2p are presented in Figure 5 and Figure 6, respectively. Curves for all gas mixtures overlap so one can conclude that the surface functionalization does not depend on the concentration of gases. The S2p peak is observed at the binding energy of approximately 169 eV what is typical for sulfites (SO_3_^2–^) [37]. Such functional groups have been observed before, even for the case of oxygen-plasma treatment of sulfur-containing polymers [44,45].

**Figure 7:**
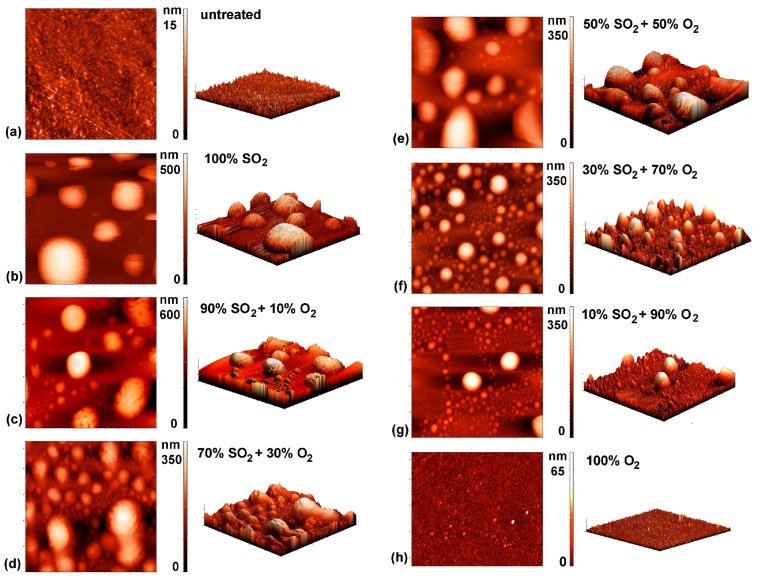
AFM images (5 × 5 µm^2^) of the samples treated in various SO_2_ + O_2_ mixtures: (**a**) untreated; (**b**) 100% SO_2_; (**c**) 10% O_2_ + 90% SO_2_; (**d**) 30% O_2_ + 70% SO_2_; (**e**) 50% O_2_ + 50% SO_2_; (**f**) 70% O_2_ + 30% SO_2_; (**g**) 90% O_2_ + 10% SO_2_; and (**h**) 100% O_2_.

More interesting are the results on the evolution of the surface morphology. Typical three-dimensional AFM images of samples treated by plasma of various gas mixtures are presented in Figure 7. The morphology of the untreated sample is presented in Figure 7a. The material is rather smooth on the micrometer scale, but the morphology of the samples treated by plasma undergoes interesting modifications.

Figure 7b represents an AFM image of the sample treated in pure SO_2_ plasma. The scale on the vertical axis is nearly 100 times larger than for the untreated sample. Nearly spherical features appeared on the polymer surface upon plasma treatment. The features are distributed randomly on the surface and the lateral dimensions are roughly a micrometer, whereas the height is somehow smaller. It appears as if droplets were formed on the surface of originally smooth material. The formation of such droplets is not typical for plasma-treated polymers. Although numerous authors reported nanostructuring of polymers upon plasma treatment [46,47,48,49,50,51,52,53,54] such a surface finish was rarely reported. The formation of such droplets cannot be explained by deposition of a third material because no other material but the polymer sample was introduced into the plasma reactor. The features were obviously formed by transformation of the polymer surface film of thickness of the order of 100 nm. Etching of polymer in plasma used in our experiments cannot cause formation of such droplets because the etching rate is just a few nm/s [55]. The formation of the droplets is rather explained by modification of the polymer structure in the surface film of thickness typical for the penetration depth of the UV radiation. As explained in the Subsection 3.1, SO_2_ plasma is a rich source of the UV radiation in the bread range from 190 to 400 nm (Figure 2). According to Kim, Ahn, and Sancaktar [56] the penetration depth of the UV radiation at wavelength of 248 and 193 nm in PET polymer is 62 and 34 nm, respectively. The penetration depth increases with increasing wavelength (decreasing photon energy). The UV radiation is absorbed in the polymer and caused a bond scission. The resulting low-mass molecular fragments rearrange according to the thermodynamic laws [57]. Since the surface energy of materials treated by oxygen-containing plasma is increased, the surface tension favorites formation of spherical features as observed in Figure 7b.

The UV radiation from plasma decreases with increasing oxygen content in the gas mixture and completely vanishes for the case of pure oxygen (Figure 3). Pure oxygen plasma does not emit UV radiation except in the VUV range where the penetration depth is minimal [58]. The morphology of the samples treated with plasma of various concentrations of gases follows the intensity of UV radiation: the droplets height decrease with the increasing oxygen content. The lateral dimension also decreases with increase of the oxygen content. The sample treated at 90% SO_2_ (Figure 7g) where the integral UV intensity is only a third of the original value (Figure 3) reveals only a few droplets of microscopic dimensions and such features vanish completely in the case of treatment with pure oxygen plasma. The sample in Figure 7h assumes morphology typical for treatment of PET in pure oxygen plasma [59]. The key modification of the PET polymers treated in plasma created in SO_2_ gases is therefore rich surface morphology what is explained by degradation of the polymer chains because of extensive UV radiation.

**Figure 8:**
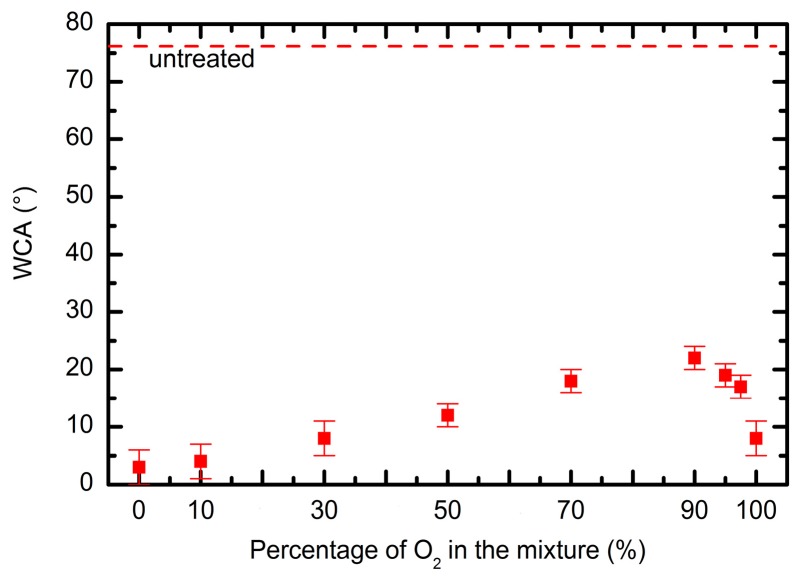
Water contact angles of the PET polymer treated in various SO_2_ + O_2_ mixtures. Zero percent of O_2_ in the mixture refers to pure SO_2_ plasma, whereas 100% of O_2_ refers to pure oxygen plasma.

Surface functionalization and a rich surface topography may have a large effect on the surface wettability. In Figure 8 are shown water contact angles for the PET polymer treated in various mixtures. A water contact angle decreased from initial ~78° to approximately 5° after the treatment in pure SO_2_ plasma. When adding oxygen to the discharge, a slight increase of the contact angle is observed to approximately 20° when the mixture of 90% O_2_ + 10% SO_2_ was used. When pure oxygen plasma was used, the contact value decreased and was approximately similar to the one obtained in pure SO_2_ plasma. Such variation of the contact angles can be explained by a different surface morphology, because the chemical state of the surface was practically similar for all the samples (Figure 4, Figure 5 and Figure 6). The influence of surface morphology on the contact angle of polymers with virtually the same surface functional groups has been already elaborated in the scientific literature [60]. There are also many other papers about tuning the wettability of materials by changing the surface roughness [61,62,63].

### 3.3. Cell Adhesion on Plasma-Treated Surfaces

**Figure 9:**
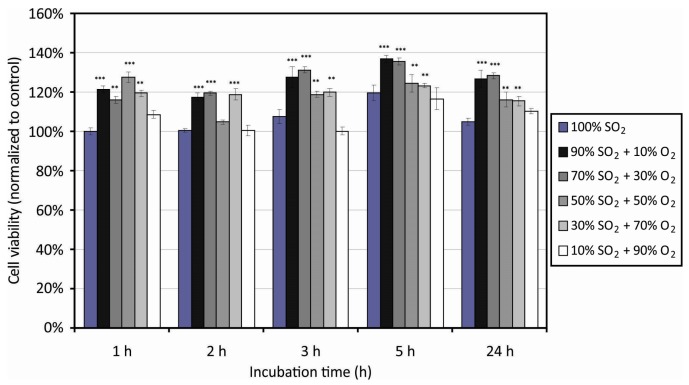
Results of the MTT assay for HUVEC cell proliferation on PET surfaces treated in various SO_2_ + O_2_ mixtures. Symbols * represent statistical significance (** represents statistical significance at *p* < 0.01 compared with the control. *** represents statistical significance at *p* < 0.001 compared with the control). Mean values (± SE) for the respective triplicates are given.

**Figure 10:**
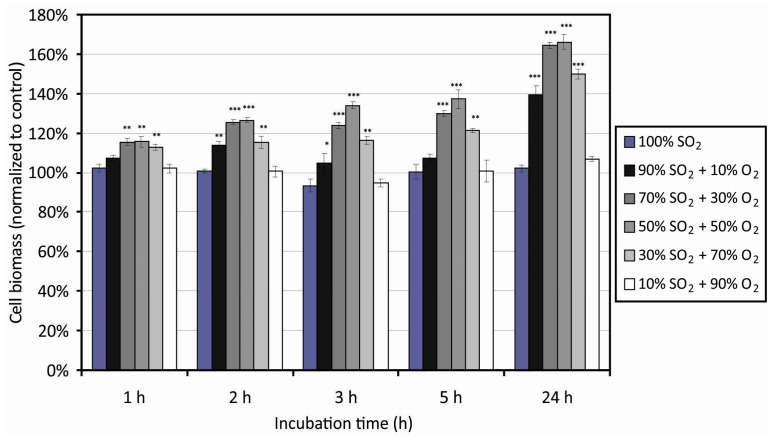
Surface cytotoxicity of PET surfaces treated in various SO_2_ + O_2_ mixtures. * represents statistical significance (* statistically significant at *p* < 0.05 compared with the control. ** represents statistical significance at *p* < 0.01 compared with the control. *** represents statistical significance at *p* < 0.001 compared with the control). Mean values ( ± SE) for the respective triplicates are given.

The polymer surface morphology and wettability may influence adhesion and proliferation of mammalian cells [64,65,66,67,68]. To reveal the influence of the morphology and wettability of PET samples functionalized with sulfate groups on the biological response, we performed systematic measurements of the cell adhesion efficiency using HUVEC cells. Biological tests were adapted to study the cell adhesion to plasma modified surfaces and their toxic effects via the metabolic activity of cells adhered to the plasma modified substrates. Figure 9 represents the results of the cellular viability using the MTT assay and Figure 10 shows the behavior of cells using the TOX test. Experiments were performed using various incubation times of 1, 2, 3, 5, and 24 h. The value of 100% was attributed to the untreated samples that served us as a control. Both figures indicate improved biocompatibility of the PET samples treated by plasma compared with the untreated ones. Differences were observed as early as one hour after cells seeding and persisted in all incubation times. After 24 h of incubation, a slight decrease in the cell viability was observed as compared to the viability after 5 h of incubation (Figure 9). At the beginning, the cells first speeded up the metabolism (ATP (adenosine triphosphate) production for synthesis of heat shock proteins and surface adhesive proteins), which was observed after 3 and 5 h of incubation as an increased cell viability. Between 5 and 24 h some cells undergo apoptosis, whereas the other cells continue growing, but slow down the metabolism to save the energy and nutrients available. Another reason for slowing down the metabolism may be toxicity of the plasma-treated samples on the adhered HUVEC cells.

The results obtained by the MTT and TOX assays are complementary—based on different experimental techniques. The MTT assay revealed metabolic activity of HUVEC adhered on the sample surface, which is directly related to cell viability. Whereas, the TOX test determined the total biomass of cells (live vs. dead) adhered on the samples, based on the intracellular proteins, detected by the test. Information on the mass of adhered cells was obtained, from what was possible to conclude on the toxic effects of the samples surface. 

**Figure 11:**
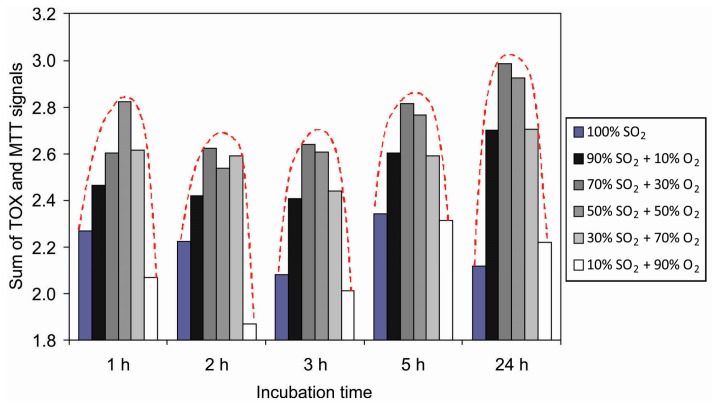
The sum of deviations from untreated samples observed for the MTT and TOX tests.

Since the results obtained by the MTT and TOX tests are scattered because of the reasons explained above, the sum of the deviations from the untreated samples is shown in Figure 11. This figure represents the sum of deviation for each treatment setting the value for the untreated sample to zero. For all treated samples there is a well-pronounced maximum in the cell proliferation versus the gas mixture. The values at various gas mixtures for particular incubation times are fitted with a parabola. The maxima for each incubation time appear between 40% and 50% of oxygen concentration. These maxima appear at moderately rough surfaces (Figure 7).

**Figure 12:**
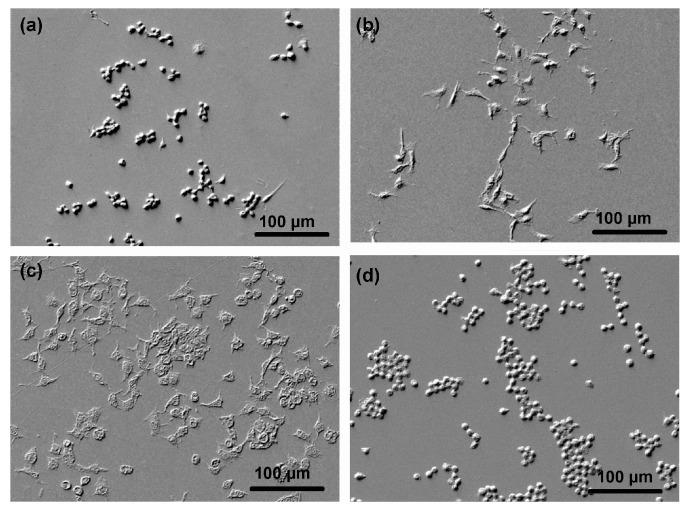
SEM images of cells on PET surfaces treated in various SO_2_ + O_2_ mixtures after 24 h of incubation: (**a**) 100% SO_2_; (**b**) 90% SO_2_ + 10% O_2_; (**c**) 60% SO_2_ + 40% O_2_; and (**d**) 10% SO_2_ + 90% O_2_.

Both MTT and TOX tests are quantitative and reveal metabolic activity and total biomass of cells seeded onto substrates. The metabolic activity and total biomass should be reflected in the morphology of cells, so we performed also SEM characterization of selected samples. This technique is qualitative because the cells are usually randomly distributed on a substrate. The most representative SEM images are shown in Figure 12. Figure 12a represents a PET sample treated with 100% SO_2_ and the cells adhered to it. As expected, the cells were clustered unevenly on the surface and have a rather circular morphology what is typical for the cells not able to fully adhere to the surface, and/or the cells responding to cytotoxic effect, indicating the apoptosis. A 10% amount of oxygen in the gas mixture allows for a surface finish which is more suitable for cell proliferation because the cells have longitudinal shape rich in long protrusions (Figure 12b). The image in Figure 12c (oxygen concentration of 40% where the MTT and TOX tests show the best results) reveals well-proliferating cells. Their spread and elongated morphology indicates good adhesion on the polymer surface. Finally, the image in Figure 12d (90% of oxygen) reveals cells of morphology similar to those on samples treated by pure SO_2_ plasma. The SEM images are therefore sound with the quantitative results obtained by the MTT and TOX tests. Rounded cluttered cells on Figure 12d are metabolically less active than spread cells on the Figure 12c, but the higher number of cells in both Figure 12c and d indicates increased biomass detected with the TOX test after 24 h of incubation. 

Given that the PET samples treated in various gas mixtures differ only in their surface morphology but not in their chemical composition, our results clearly show that the surface morphology of plasma-treated polymers has an important influence on cell adhesion and proliferation. Regarding the surface wettability of the plasma-treated samples, it seems that it does not have an important influence on the cell adhesion in our case, because changes in the surface wettability are not so pronounced to explain changes in the cell adhesion and proliferation. Furthermore, a trend of monotonous increase of the contact angles with the increasing oxygen content in the mixture is not observed in the case of cell proliferation.

## 4. Conclusions

The combination of various experimental biological techniques allowed for studying the behavior of HUVEC cells seeded on PET substrates which had been previously modified by gaseous plasma created in various mixtures of sulfur dioxide and oxygen gases. The best proliferation was observed in the case of nearly the same amounts of both mentioned gasses. The key reason for better proliferation of this type of human cell is likely due to the appropriate structuring of the substrate morphology of the sulfurized polymer surface. The best improvement of the polymer biocompatibility was obtained in the gas mixtures of approximately 60% SO_2_ where the surface of the sulfonated polymer was covered with nearly spherical structures of lateral dimension of approximately 1 micrometer. The rich morphology of the samples treated in gaseous plasma with substantial concentration of sulfur dioxide was explained by modification of the polymer within the surface film of several 100 nm. The modification was due to the bond scission of this type of polymer caused by the ultraviolet radiation.
